# The Use of Complementary and Alternative Medicine Is Less Frequent in Patients with Inflammatory Bowel Disease Than in Patients with Other Chronic Gastrointestinal Disorders

**DOI:** 10.1155/2018/9137805

**Published:** 2018-04-03

**Authors:** Anna Fábián, Mariann Rutka, Tamás Ferenci, Renáta Bor, Anita Bálint, Klaudia Farkas, Ágnes Milassin, Kata Szántó, Zsuzsanna Lénárt, Ferenc Nagy, Zoltán Szepes, Tamás Molnár

**Affiliations:** ^1^First Department of Medicine, University of Szeged, Korányi fasor 8-10, Szeged 6720, Hungary; ^2^John von Neumann Faculty of Informatics, Óbuda University, Bécsi út 96b, Budapest 1034, Hungary

## Abstract

**Background and Aims:**

Complementary and alternative medicine (CAM) is commonly used among patients with inflammatory bowel diseases (IBD), but evidence about its real-life use is limited. We aimed to assess and compare CAM use in outpatients with IBD and other gastrointestinal diseases.

**Materials and Methods:**

The use of herbs and botanicals, lifestyle modifications and mind/body therapies, patient satisfaction, and continuous use of conventional medicine were assessed with an anonymous questionnaire at a tertiary IBD unit in Hungary. 396 IBD patients (207 with Crohn's disease, 185 with ulcerative colitis, and 4 with indeterminate colitis) and 164 patients with gastric acid-related diseases, premalignant and malignant colorectal diseases, lactose intolerance, celiac disease, dysbacteriosis, and so on were included.

**Results:**

IBD patients reported significantly lower usage of herbs than did controls (25% versus 42%, *p* < 0.001). More than 90% of responding IBD patients continued conventional medication besides herbal remedies (83% in unaltered doses). IBD patients were more likely to implement lifestyle modifications (77% versus 63%, *p* = 0.0011), but not body/mind therapies (20% versus 15%, *p* = 0.1516). Younger age was a significant predictor of lifestyle modifications (*p* = 0.0246).

**Conclusions:**

CAM use (especially that of herbal remedies) in IBD is less frequent than that in other gastrointestinal diseases. It is more a complementary than an alternative to conventional medicine in IBD. There is no significant difference between CAM use in patients with Crohn's disease and that in patients with ulcerative colitis, although the latter tend to choose herbs; the benefit of which is supported by scientific evidence. This study is registered at the Medical Research Council, Hungary. This trial is registered with 3769/2010/1018EKU.

## 1. Introduction

Inflammatory bowel diseases (IBD) including Crohn's disease (CD) and ulcerative colitis (UC) have a significant impact on health-related quality of life [1]. Besides the debilitating symptoms of relapses, psychological distress associated with unpredictable disease course and development of complications and adverse events related to medication often occurs during remission [2, 3]. The desire to gain more control over IBD and to be treated as a whole person might lead patients to unconventional treatment methods.

Complementary and alternative medicine (CAM) is an umbrella term for a set of health care practices that are not part of a country's traditional medical practices and are not integrated into the dominant health care system. Generally, alternative medicine refers to methods that replace traditional treatments, while complementary medicine involves those that are used as an addition to conventional therapy. CAM use seems to be increasing in recent decades. According to a recent survey, it is around 40% in chronic gastrointestinal conditions and varies between 20 and 60% in IBD as a special subgroup of the previous [3–7].

The aim of this study was to examine the frequency and predictors of regular CAM use in IBD patients and to compare data with the one obtained from patients diagnosed with other chronic gastrointestinal diseases.

## 2. Methods

An anonymous questionnaire was distributed to outpatients at a tertiary IBD unit in Szeged, Hungary, between February and October 2015. Patients were categorized as suffering from IBD (CD, UC, and indeterminate colitis) or any other chronic gastrointestinal disease (control group: gastric acid-related diseases [reflux disease, gastric or duodenal ulcer, etc.], irritable bowel syndrome, celiac disease, lactose intolerance, colorectal diseases [e.g., diverticulosis or malignancies], and other). The survey focused on the use of herbs and botanicals, lifestyle modifications (exercise, diet, or cessation of smoking), and mind/body therapies (stress management, relaxation techniques [autogenic training, brain control, meditation, and hypnotherapy], massage, kinesiology, yoga, acupuncture, etc.). Frequency of CAM use, patient satisfaction, and continuous use of conventional medicine were assessed in each category. The study was registered at the Medical Research Council, Hungary, with the registration identification number 3769/2010/1018EKU and was conducted in accordance with the principles of the Declaration of Helsinki.

### 2.1. Statistical Analysis

Categorical variables are presented as percentages and are compared among groups using Fisher's exact test. Multivariate analysis to investigate predictors of CAM usage was performed with penalized logistic regression, controlling for age, sex, concurrent conventional medication, and disease duration. Age and disease duration were entered into the model with restricted cubic spline expansion to allow for a flexible functional form; however, no interaction was allowed between the variables. The necessity of nonlinearity was checked with a joint *F*-test on nonlinear terms (prespecified test), and a linear model was specified if *p* < 0.05 in this test. Penalty was chosen to optimize Hurvich and Tsai's corrected AIC [8]. Statistical analysis was performed under R program package version 3.3.2 [9] with a custom script that is available at the corresponding author on request using library rms version 5.1-0 [10].

## 3. Results

**Figure 1:**
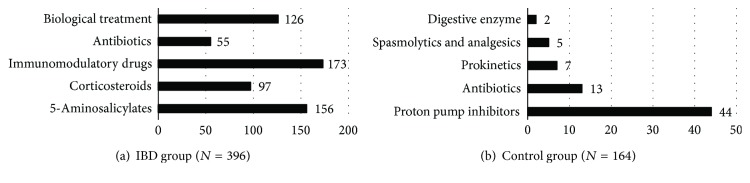
Concurrent medication in the IBD (a) and control group (b). IBD: inflammatory bowel diseases.

396 consecutive IBD patients (207 with CD, 185 with UC, and 4 with indeterminate colitis; mean age: 42 years; male/female ratio: 183/205 ([8] patients gave no answer); mean disease duration: 11 years) and 164 patients with other chronic gastrointestinal diseases (gastric acid-related diseases [*N* = 56], premalignant and malignant colorectal diseases [*N* = 33], diverticulosis and irritable bowel syndrome [*N* = 22], lactose intolerance [*N* = 11], celiac disease [*N* = 12], and dysbacteriosis and other [*N* = 30]; mean age: 53 years; male/female ratio: 40/124; mean disease duration: 5 years) were included in our study. 92% of IBD patients (*N* = 364) were taking medication (5-aminosalicylates, corticosteroids, immunomodulatory drugs, antibiotics, or biologics) for their IBD at the time of the survey, while only 35% of control patients (*N* = 58) were on drugs (proton pump inhibitors, antibiotics, prokinetics, spasmolytic and analgesic drugs, and digestive enzymes) (Figure 1).

Total CAM use (including any of herbs/botanicals, lifestyle changes, and mind/body therapies) was 80% among IBD patients and 74% in the control group (*p* = 0.141). Almost two-thirds of CAM users applied at least two methods simultaneously (62% and 65% for IBD and control patients, resp.) (range: 1–9). In the IBD group, there was no significant difference between total CAM use of patients with CD and that of patients with UC (79% and 83%, *p* = 0.4407). There was no significant difference between the two phenotypes of IBD regarding the use of herbal remedies (*p* = 0.1033), lifestyle modifications (*p* = 1), or body/mind therapies (*p* = 0.6147).

### 3.1. Herbs and Botanicals

**Figure 2:**
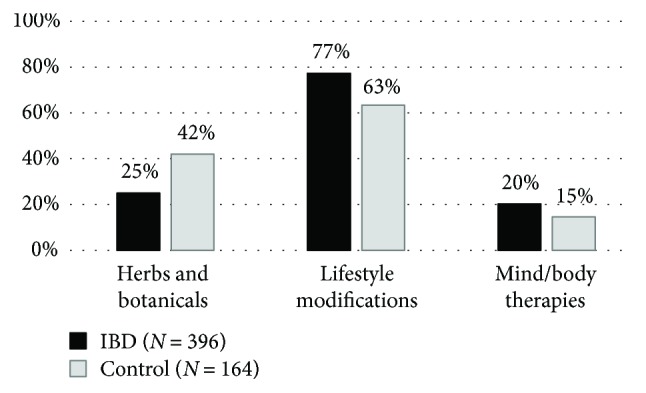
Frequency of CAM use in the IBD and control group. CAM: complementary and alternative medicine; IBD: inflammatory bowel diseases.

**Figure 3:**
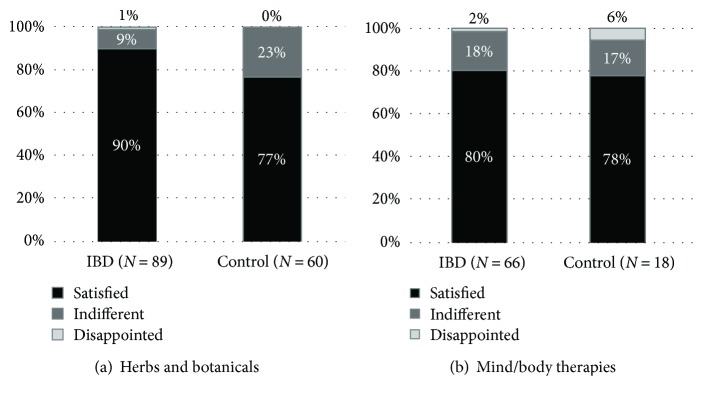
Patient opinions about herbs and botanicals (a) and mind/body therapies (b). IBD: inflammatory bowel diseases.

**Figure 4:**
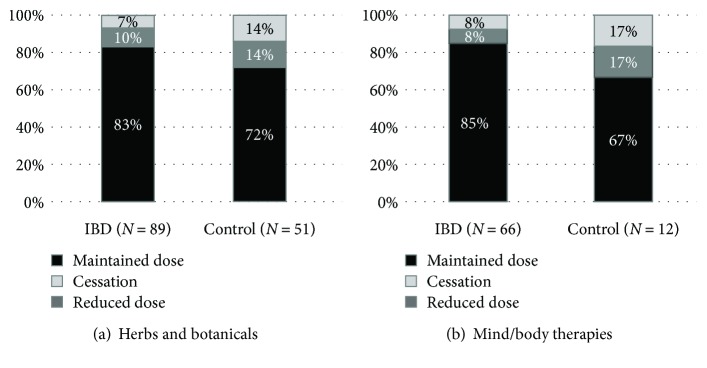
Continuation rates of conventional medication among patients using (a) herbs and botanicals and (b) mind/body therapies. IBD: inflammatory bowel diseases.

IBD patients reported significantly lower use of herbal remedies than did controls (25% versus 42%, *p* < 0.001) (Figure 2) and were more likely to use a single herbal product (62% versus 52%). *Aloe vera* was the most popular in both groups: 24% and 32% of those administering herbs reported its use (Table 1). The majority of patients were satisfied with the products (Figure 3(a)). More than 90% of IBD patients continued their conventional medication, and 83% of the responders did it so by maintaining the original dose. Continuation rates of conventional therapies were similar, although somewhat lower in the control group (Figure 4(a)).

**Figure 5:**
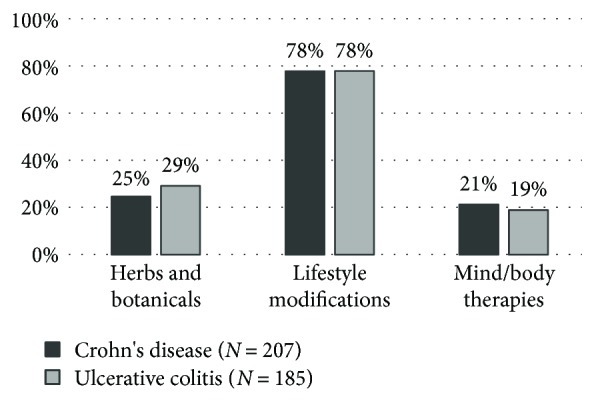
Frequency of CAM use among IBD patients. CAM: complementary and alternative medicine; IBD: inflammatory bowel diseases.

**Figure 6:**
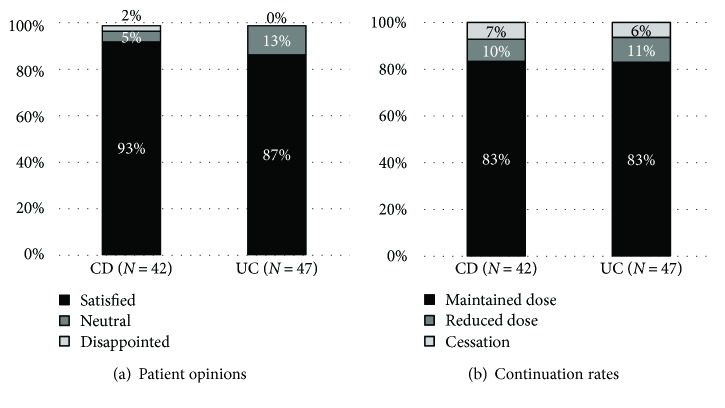
Patient opinions about herbs and botanicals (a) and continuation rates of conventional medication (b) among IBD patients using herbal products. IBD: inflammatory bowel diseases; CD: Crohn's disease; UC: ulcerative colitis.

Usage rates of herbs and botanicals were similar in CD and UC patients (25% and 29%), with nearly two-thirds of them administering a single product (63% and 61%, resp.) (Figure 5). *Aloe vera* was the most commonly used in both IBD groups (18% of CD patients and 28% of UC patients reported its use). Interestingly, milk thistle was almost five times as popular among patients with UC as among those with CD (Table 1). Generally, both CD and UC patients considered herbal remedies beneficial, and only one patient with Crohn's disease reported aversion towards their use (Figure 6(a)). A total of 6 IBD patients reported cessation of conventional medication (3 patients with CD and 3 patients with UC), and 83% of patients continued with the conventional IBD therapy in both groups (Figure 6(b)).

### 3.2. Lifestyle Modifications

IBD patients were more likely to implement lifestyle modifications after the diagnosis compared to the control group (77% versus 63%, *p* = 0.0011) (Figure 2). The high rate was mainly attributable to dietary changes in both groups (70% versus 57% in the IBD group and control group, resp.). More than 20% of patients started regular exercise (29% versus 20%), and more than 7% of them stopped smoking. Interestingly, three times as many patients with CD as those with UC quitted smoking (30 versus 11). No such difference could be observed between IBD subgroups regarding dietary changes and exercise (Table 1). None of the patients reported aversion to lifestyle changes, and only 15 IBD (9 with CD and 6 with UC) and 2 control patients were neutral.

### 3.3. Mind and Body Therapies

20% of IBD and 15% of control patients (*p* = 0.1516) used mind/body therapies (Figure 2). In both groups, patients preferred relaxation techniques the most (Table 1) and were likely to stick with one technique at a time (68% and 58% for the IBD group and control group, resp.). 80% and 78% of the responders in each group were satisfied with the applied mind/body therapy, and only two patients (one with CD and one with reflux disease) reported negative opinion about stress management techniques (Figure 3(b)). Only five IBD (3 with CD and 2 with UC) patients stopped their conventional medication after starting a mind/body technique, and 85% of the responders continued with traditional IBD therapy in unchanged doses. This rate was only 67% for the control group, but a low case number may prevent reliable assessment (Figure 4(b)).

### 3.4. Multivariate Analysis of Predictive Factors

Patients with indeterminate colitis were not investigated in the multivariate model due to their low count. In the multivariate model, neither female gender (*p* = 0.0763), nor younger age (*p* = 0.3326), nor disease duration (*p* = 0.4227) predicted the use of herbs and botanicals; however, IBD patients were significantly less likely to use this modality (OR = 0.58 [95% CI: 0.38–0.88], *p* = 0.0097). Younger age was found to be a significant predictor of lifestyle modification (*p* = 0.0246), but not the usage of mind/body therapies (*p* = 0.3425). None of female gender, disease duration, or IBD predicted the use of these CAMs (*p* = 0.6295, *p* = 0.0847, and *p* = 0.1172 for lifestyle changes and *p* = 0.5825, *p* = 0.3018, and *p* = 0.5246 for mind/body therapies). In an extended model for the IBD group that included disease phenotype, steroid intake, and usage and kind of biological therapy in addition to age, sex, and disease duration, none was found to be associated with herbal therapy, lifestyle modification, or mind/body therapies.

## 4. Discussion

CAM use is around 50% in developed countries and over 80% in underdeveloped countries [11] with a substantial rise in Europe over the last two decades [12]. In case of chronic gastrointestinal conditions, especially functional disorders, this rate is around 40% [5]. In IBD, a special subgroup of the above, it varies between 20% and 60%, and occasional CAM use might be as high as 81% [3, 4, 6, 7, 13]. Variations in usage rates and the most common CAM types might be attributable to ambiguous definition (e.g., acupuncture is considered CAM in Europe, whereas it is a traditional method in Asia) and inconsistent inclusion criteria of CAM (e.g., vitamins, exercise, and prayer) [4, 14]. These variations are reflected in our study too; usage rates of different CAM types showed significant differences in both groups. Given the fact that multiple CAM use is a common phenomenon [4, 6], CAM options can hardly be evaluated on their own.

### 4.1. Herbs and Botanicals

Herbal remedies are among the most popular and well-studied CAM options. According to a Canadian nationwide survey among IBD patients, herbs were most frequently administered (41%) [4]. A recent meta-analysis provided evidence about the efficacy of anti-inflammatory *Aloe vera* in UC of mild-to-moderate activity, the beneficial effects of wheat grass juice in proctitis, and the feasibility of curcumin and *Plantago ovata* in maintaining remission in UC (the latter was reported to be of similar efficacy as mesalazine) [15]. According to a recent review of controlled trials investigating herbal products in IBD, *Andrographis paniculata* (Indian Echinacea), *Boswellia serrata*, and topical Xilei-San might also be useful in active UC, and a mixture of myrrh, chamomile flower extract, coffee charcoal, and milk thistle can also be beneficial in inactive UC for maintaining remission [16]. Regarding CD, a smaller number of clinical trials were conducted, most of them with poor design and small case number. Nevertheless, wormwood and *Boswellia serrata* showed promising results for active CD, and Tripterygium wilfordii can have a potential benefit in inactive CD for the prevention of relapses [15, 16]. Our study revealed that there was no significant difference between the types of herbs used by UC and CD patients, although more UC than CD patients chose *Aloe vera*, milk thistle, curcumin, and wheatgrass. This might reflect potential patient awareness of the beneficial effect of these products on UC. On the other hand, no use of wormwood, *Boswellia serrata*, or Tripterygium wilfordii was reported by CD patients, suggesting less awareness of these herbs. However, this fact did not seem to alter patient satisfaction with the administered herbal product(s), nor continuation rates of conventional IBD medication (Figure 6).

It should be highlighted that reliable data on the efficacy of herbs is still limited, and potential adverse events and interactions with conventional medications should also be noted [3]. Although the most commonly used herbal products in our study (Table 1)—except for medicinal fungi—are not among those reported with a hepatotoxic effect [17], health care professionals should be aware of the potential hepatotoxicity of herbs, especially in the case of IBD-associated liver diseases.

The significantly lower use of herbs in IBD compared to other gastrointestinal conditions—considering the high usage and continuation rates of traditional IBD medications as well—suggests remarkable trust in conventional therapy and more profound knowledge about the disease and also reflects fear of relapses and complications. Our study was conducted in a tertiary IBD center; hence, patient selection can possibly lead to biases as patients attending specialized clinics tend to have more trust in conventional medicine compared to the general population [4]. According to Nguyen et al., only CAM applied for IBD treatment (and not for the general well-being) impairs adherence to traditional medication [18].

### 4.2. Lifestyle Changes

It is debatable whether lifestyle changes should be defined as CAM, but our patients were most likely to rank them so. As the potential benefit of CAM lies mostly in the improved sense of disease control [19], patients might gain even greater sense of control altering those aspects of life that are “untouchable” to conventional IBD therapies.

A high-protein diet may be associated with an increased risk of IBD, while fruit and vegetable intake might decrease it [20]. Although evidence on the benefit of dietary modifications in IBD is limited, according to Zallot et al., 58% of IBD patients believed in the role of diet in relapses and were prone to avoid certain foods [21]. A Finnish study comparing adolescents with IBD and juvenile idiopathic arthritis reported self-imposed dietary restrictions in 64.8% of CAM users [13].

Moderate physical activity might complement conventional IBD therapy. Besides improving the individual's general well-being and fitness level, regular exercise may also have beneficial effects on immunological response, psychological health, nutritional status, and bone mineral density. Studies suggest potential anti-inflammatory effects of myokines released during skeletal muscle contractions, but further investigation is needed to clarify the exact mechanisms [22].

While smoking is generally considered a major environmental risk factor for multiple diseases including vascular disease, various neoplasia, and chronic obstructive pulmonary disease, evidence proves that smoking cessation might exacerbate disease activity and symptoms in UC [23]. On the other hand, it has the opposite effect on CD, as quitting smoking generally results in decreased disease activity [24]. These effects are also reflected in our results: CD patients were more likely to quit smoking than UC patients.

### 4.3. Mind/Body Therapies

Besides the psychological burden associated with IBD (including but not limited to stress in intimate relationships, worrying over disease complications, depression, and embarrassment), perceived—especially prolonged—stress is a significant predictor for relapses [25, 26]. Brief positive effects were reported for health-related quality of life; nevertheless, identifying the optimal target of mind/body therapies is also an issue. Berrill et al. defined irritable bowel syndrome-type symptoms in IBD as potential therapeutic targets [27]. Jedel et al. identified a subgroup of patients with higher stress levels that benefited from mind/body therapies in terms of disease activity [28]. Despite the promising short-term results regarding relaxation techniques, evidence is lacking about the feasibility of mind/body therapies in IBD as maintenance treatment or prevention of relapses [29, 30].

### 4.4. Predictors of CAM Usage

Previous studies associated female gender, low level of confidence in the physician, and research of disease-related information with CAM usage, but no role of disease activity or severity could be determined [6]. Portela et al. defined steroid prescription (*p* < 0.001) and higher education level (*p* = 0.003) as predictors of CAM use [7]. According to a population-based case control study from New Zealand, female gender (*p* < 0.001), younger age (*p* = 0.005), higher education (*p* = 0.002), higher income (*p* = 0.04), being a vegetarian (*p* < 0.001), and a middle social class at birth (*p* = 0.024) were independent predictors of oral CAM use in IBD unlike disease phenotype [31]. Our study confirmed younger age as a predictive factor of lifestyle modifications, but female gender, longer disease duration, disease phenotype, and type and number of conventional medications were not associated with CAM use.

## 5. Limitations

The survey was conducted at a tertiary IBD center; thus, data is not representative of the general population. The control group of chronic gastrointestinal conditions other than IBD was rather heterogeneous, and this might result in slight biases. Cross-sectional design was also a limiting factor, as well as the fact that not all patients responded to all survey questions. The survey did not include patient satisfaction with conventional medication, nor self-evaluated severity assessment of the disease.

## 6. Conclusions

Our study revealed that CAM use is relatively common among IBD patients, especially in terms of lifestyle modification (predominantly dietary changes and exercise). Usage rates of herbs and botanicals were significantly lower among patients with other chronic gastrointestinal disorders, and IBD patients tended to be more adherent to traditional medication, potentially suggesting a higher level of disease awareness and trust in conventional remedies. Application rates of lifestyle modifications and mind/body therapies were similar in IBD and other gastrointestinal diseases.

Patients with UC tended to administer herbal products; the beneficial effect of which is supported by scientific evidence, whereas no such tendency could be observed in patients with CD, possibly suggesting less awareness of potentially useful herbal remedies for CD. Nevertheless, this did not alter patient satisfaction or adherence to conventional IBD therapy.

High adherence rates to conventional therapy may suggest that patients prefer to use CAM as an adjunction rather than as a replacement to traditional medicine. However, there is a need for further studies with a homogenized large case number investigating the frequency and characteristics of CAM use in IBD and clarifying the potentially different CAM choices of patients with CD and UC. Still, considering the high frequency and multiple choices of CAM, physicians and nurses involved in IBD care should not only be aware of the most common knowledge about CAM but also be able to provide appropriate information and guidance to patients in order to develop high-quality care.

## Figures and Tables

**Table 1:** The most preferred CAM types.

	IBD^∗^ (*N* = 396)	CD (*N* = 207)	UC (*N* = 185)	Control group (*N* = 164)
Herbs and botanicals				
*Aloe vera*	24	9	15	22
Milk thistle	11	2	9	13
Walnut leaf	11	7	4	0
Curcumin	10	4	6	14
Wheatgrass	9	3	6	9
*Plantago ovata*	7	3	4	4
Medicinal fungi	7	3	4	1
Chamomile	6	5	1	4
Lifestyle changes				
Special diet	279	146	133	94
Exercise	112	64	48	32
Cessation of smoking	41	30	11	12
Mind/body therapies				
Relaxation techniques	36	18	17	16
Stress management	28	16	12	7
Kinesiology, yoga, massage	23	15	7	7
Acupuncture	16	7	8	4

∗ includes patients with Crohn's disease (CD), ulcerative colitis (UC), and indeterminate colitis. CAM: complementary and alternative medicine; IBD: inflammatory bowel diseases.

## Supplementary Materials

Supplementary Materials STROBE statement for observational studies.

## References

[1] Pizzi L. T., Weston C. M., Goldfarb N. I. (2006). Impact of chronic conditions on quality of life in patients with inflammatory bowel disease. *Inflammatory Bowel Diseases*.

[2] Becker H. M., Grigat D., Ghosh S. (2015). Living with inflammatory bowel disease: a Crohn’s and Colitis Canada survey. *Canadian Journal of Gastroenterology and Hepatology*.

[3] Langhorst J., Wulfert H., Lauche R. (2015). Systematic review of complementary and alternative medicine treatments in inflammatory bowel diseases. *Journal of Crohn's & Colitis*.

[4] Hilsden R. J., Verhoef M. J., Best A., Pocobelli G. (2003). Complementary and alternative medicine use by Canadian patients with inflammatory bowel disease: results from a national survey. *The American Journal of Gastroenterology*.

[5] Dossett M. L., Davis R. B., Lembo A. J., Yeh G. Y. (2014). Complementary and alternative medicine use by US adults with gastrointestinal conditions: results from the 2012 National Health Interview Survey. *The American Journal of Gastroenterology*.

[6] Bensoussan M., Jovenin N., Garcia B. (2006). Complementary and alternative medicine use by patients with inflammatory bowel disease: results from a postal survey. *Gastroentérologie Clinique et Biologique*.

[7] Portela F., Dias C. C., Caldeira P. (2017). The who-when-why triangle of complementary and alternative medicine use among Portuguese IBD patients. *Digestive and Liver Disease*.

[8] Harrell F. (2015). *Regression Modeling Strategies: With Applications to Linear Models, Logistic and Ordinal Regression, and Survival Analysis*.

[9] R Core Team (2016). *R: A Language and Environment for Statistical Computing*.

[10] Harrell F. E. rms: regression modeling strategies. R package version 5.1-0. https://CRAN.R-project.org/package=rms.

[11] Ishaque S., Saleem T., Qidwai W. (2009). Knowledge, attitudes and practices regarding gemstone therapeutics in a selected adult population in Pakistan. *BMC Complementary and Alternative Medicine*.

[12] Fischer F. H., Lewith G., Witt C. M. (2014). High prevalence but limited evidence in complementary and alternative medicine: guidelines for future research. *BMC Complementary and Alternative Medicine*.

[13] Nousiainen P., Merras-Salmio L., Aalto K., Kolho K. L. (2014). Complementary and alternative medicine use in adolescents with inflammatory bowel disease and juvenile idiopathic arthritis. *BMC Complementary and Alternative Medicine*.

[14] Rawsthorne P., Shanahan F., Cronin N. C. (1999). An international survey of the use and attitudes regarding alternative medicine by patients with inflammatory bowel disease. *The American Journal of Gastroenterology*.

[15] Triantafyllidi A., Xanthos T., Papalois A., Triantafillidis J. K. (2015). Herbal and plant therapy in patients with inflammatory bowel disease. *Annals of Gastroenterology*.

[16] Sebepos-Rogers G. M., Rampton D. S. (2017). Herbs and inflammatory bowel disease. *Gastroenterology Clinics of North America*.

[17] Abdualmjid R. J., Sergi C. (2013). Hepatotoxic botanicals – an evidence-based systematic review. *Journal of Pharmacy & Pharmaceutical Sciences*.

[18] Nguyen G. C., Croitoru K., Silverberg M. S., Steinhart A. H., Weizman A. V. (2016). Use of complementary and alternative medicine for inflammatory bowel disease is associated with worse adherence to conventional therapy: the COMPLIANT study. *Inflammatory Bowel Diseases*.

[19] Hilsden R. J., Scott C. M., Verhoef M. J. (1998). Complementary medicine use by patients with inflammatory bowel disease. *The American Journal of Gastroenterology*.

[20] Hou J. K., Abraham B., El-Serag H. (2011). Dietary intake and risk of developing inflammatory bowel disease: a systematic review of the literature. *The American Journal of Gastroenterology*.

[21] Zallot C., Quilliot D., Chevaux J. B. (2013). Dietary beliefs and behavior among inflammatory bowel disease patients. *Inflammatory Bowel Diseases*.

[22] Bilski J., Brzozowski B., Mazur-Bialy A., Sliwowski Z., Brzozowski T. (2014). The role of physical exercise in inflammatory bowel disease. *BioMed Research International*.

[23] Bastida G., Beltrán B. (2011). Ulcerative colitis in smokers, non-smokers and ex-smokers. *World Journal of Gastroenterology*.

[24] Rosenfeld G., Bressler B. (2012). *Editorial*: the truth about cigarette smoking and the risk of inflammatory bowel disease. *The American Journal of Gastroenterology*.

[25] Bernstein C. N., Singh S., Graff L. A., Walker J. R., Miller N., Cheang M. (2010). A prospective population-based study of triggers of symptomatic flares in IBD. *The American Journal of Gastroenterology*.

[26] Levenstein S., Prantera C., Varvo V. (2000). Stress and exacerbation in ulcerative colitis: a prospective study of patients enrolled in remission. *The American Journal of Gastroenterology*.

[27] Berrill J. W., Sadlier M., Hood K., Green J. T. (2014). Mindfulness-based therapy for inflammatory bowel disease patients with functional abdominal symptoms or high perceived stress levels. *Journal of Crohn's & Colitis*.

[28] Jedel S., Hoffman A., Merriman P. (2014). A randomized controlled trial of mindfulness-based stress reduction to prevent flare-up in patients with inactive ulcerative colitis. *Digestion*.

[29] Langhorst J., Mueller T., Luedtke R. (2007). Effects of a comprehensive lifestyle modification program on quality-of-life in patients with ulcerative colitis: a twelve-month follow-up. *Scandinavian Journal of Gastroenterology*.

[30] Elsenbruch S., Langhorst J., Popkirowa K. (2005). Effects of mind-body therapy on quality of life and neuroendocrine and cellular immune functions in patients with ulcerative colitis. *Psychotherapy and Psychosomatics*.

[31] Koning M., Ailabouni R., Gearry R. B., Frampton C. M. A., Barclay M. L. (2013). Use and predictors of oral complementary and alternative medicine by patients with inflammatory bowel disease: a population-based, case–control study. *Inflammatory Bowel Diseases*.

